# Pharmacists’ Attitudes, Perceptions, and Preferences Regarding Continuing Education: Cross-Sectional Study in Vietnam

**DOI:** 10.2196/77013

**Published:** 2025-12-16

**Authors:** Trung Quang Vo, Phuoc Duy Le, Hien Thi Bich Tran, Hieu Thi Thanh Nguyen, Thoai Dang Nguyen, Trang Nguyen Khanh Huynh, Bay Van Vo

**Affiliations:** 1 Department of Economic and Administrative Pharmacy, Faculty of Pharmacy, Pham Ngoc Thach University of Medicine, No 2. Duong Quang Trung, Hoa Hung Ward, Hochiminh City, 700000, Vietnam, (+84) 283865243; 2 Social, Economic and Administrative Pharmacy (SEAP) Graduate Program, Faculty of Pharmacy, Mahidol University, Bangkok, Thailand; 3 Department of Obstetrics and Gynecology, Faculty of Medicine, Pham Ngoc Thach University of Medicine, Hochiminh City, Vietnam

**Keywords:** attitudes, continuing education, pharmacist, perception, preferences, Vietnam

## Abstract

**Background:**

The evolution of the health care landscape necessitates expanding the roles of pharmacists in patient-centered care to encompass direct patient management, collaborative practice, and preventive service. These responsibilities can be fulfilled by pharmacists through ongoing professional development, in which continuing education (CE) is instrumental to career advancement and improved patient care.

**Objective:**

This cross-sectional study aimed to assess Vietnamese pharmacists’ attitudes, perceptions, and preferences regarding CE.

**Methods:**

Participants were recruited via convenience and snowball sampling, after which a validated 42-item questionnaire was administered to them through online and offline channels from December 2024 to February 2025. The data were examined via descriptive statistical analysis using SPSS (version 26.0; IBM Corp). The associations between participant characteristics and attitudinal or perception scores (*P*<.05) were assessed using 1-way ANOVA with 1000 bootstrap samples.

**Results:**

This study involved 508 pharmacists, most of whom were aged 25 to 30 years (n=197, 38.8%), and the majority held university degrees (n=360, 70.9%). Their mean attitudinal score was 44.4 (SD 5.5), reflecting generally positive attitudes toward CE. However, significant differences in mean attitudinal scores were found across groups categorized by education level, job position, and frequency of overtime (*P*<.05). More than half of the participants derived good scores on their perceptions of CE, with their preferred CE formats including computer- and internet-based learning, as well as the use of medical search engines. Finally, the pharmacists expressed a strong preference for CE topics focusing on skill development.

**Conclusions:**

The Vietnamese pharmacists exhibited positive attitudes toward CE, favoring flexible learning formats and practical topics. These insights can inform the efforts of policymakers and educators to enhance CE accessibility, improve pharmacists’ competencies, and, ultimately, advance patient care.

## Introduction

Continuing education (CE) is defined by the World Health Organization (WHO) as the process of updating professional knowledge after the completion of formal education, enabling health care professionals to maintain and enhance their clinical practice [1]. In 2015, the Accreditation Council for Pharmacy Education expanded its CE framework by introducing the concept of continuing professional development (CPD), which is characterized as a self-directed, ongoing, systematic, and goal-oriented learning process embedded within professional practice [2]. CPD is to describe the broader, holistic process by which health professionals maintain and enhance their knowledge, skills, and performance throughout their careers [2]. This encompasses not only formal learning activities but also self-directed learning, quality improvement initiatives, and mentorship. CE, in contrast, refers specifically to structured, formal educational activities, such as courses, workshops, and conferences, which are a subset of CPD [2,3]. In Vietnam, the Ministry of Health has enacted CE policies since 2013 through Circular No. 22/2013/TT-BYT, later amended through Circular No. 26/2020/TT-BYT [4]. The Vietnamese primary framework for lifelong learning is designated as CE, which includes various short-term training programs such as professional development courses, continuing medical education, and technology transfer training [5]. From a broader international perspective, this CE framework constitutes a central component of CPD, which encompasses a wider range of formal and informal learning activities. Circular No. 32/2023/TT-BYT requires a minimum of 120 CE credit hours over 5 years from the medical practitioners [6], while Decree No. 163/2025/NĐ-CP, which amends the Law on Pharmacy No. 105/2016/QH13 [7], mandates the completion of at least 8 credit hours every three years by pharmacists [8]. These regulatory distinctions reflect a progressive shift toward role-specific CE requirements for different health care professionals. However, the absence of a globally standardized definition of CE brings about variations in CE awareness, structural implementation, and regulatory frameworks across countries. While the policy framework is being constructed, there is a striking lack of empirical research into how Vietnamese pharmacists themselves perceive and experience this evolving CE system.

CE is pivotal in ensuring the continued competence of health care professionals, ultimately contributing to improved patient care and efficiency in health care systems. Among such professionals, pharmacists need to take on roles that go beyond traditional dispensing duties to include direct patient care, collaborative practice, comprehensive medication management, and preventive care service [9]. This means that CE is essential to updating clinical knowledge and skills. CE is critical for maintaining excellent pharmacy practice and optimizing health care outcomes. Its importance grows as technology advances, evidence-based medicine expands, and health care becomes increasingly globalized. CE is also a prerequisite for the renewal of pharmacist licenses, with specific credit requirements enforced in jurisdictions such as France and the United States. Similarly, the United Kingdom and Canada implement a mandatory CPD approach [10]; European countries, such as Belgium and Norway, link CE completion to salary increments; and the Netherlands, Austria, Switzerland, Spain, Hungary, and Italy treat CE as compulsory for health care professionals [11]. The same holds true for Vietnam, where pharmacists must fulfill CE requirements within 3 years of obtaining their practice certificates to maintain licensure [7].

Accordingly, assessing pharmacists’ attitudes, perceptions, and preferences regarding CE is crucial for optimizing training programs and ensuring their relevance to professional practice. In this context, identifying cognitive gaps—defined as the discrepancies between pharmacists’ current knowledge, skills, or perceptions and the expected competencies—serves a dual role. First, it enhances the evaluation of attitudes by revealing potential misconceptions or underrecognized needs that may influence learning motivation. Second, it informs how CE programs are designed and delivered. This ensures content and instructional strategies match real-world professional demands and learners’ expectations. Several studies have examined these variables in relation to CE participation, particularly in Gulf and Middle Eastern countries [12-18]. These studies demonstrated that most pharmacists recognize CE as essential for professional development. Research in Kuwait indicated that over 60% of pharmacists exhibit positive attitudes toward CE, with workshops being the most preferred modality [16]. Enhancing pharmacists’ engagement with CE requires the provision of educational content in preferred formats, such as interactive workshops or structured discussions, to improve knowledge retention and application [12]. Meanwhile, a study in Ethiopia found that 56.5% of pharmacists are unfamiliar with the concept of CE, highlighting regional disparities in CE awareness and accessibility [19].

Despite the growing importance of CE, limited evidence has been derived as to Vietnamese pharmacists’ attitudes, perceptions, and preferences regarding CE. To address this deficiency, this study evaluated the aforementioned variables to contribute to essential endeavors to strengthen the national CE system in the country and improve pharmacists’ access to CE opportunities. The findings will provide the first crucial evidence to inform the effective development of the national CE system, ensuring it is responsive to its end users. From a global perspective, this research offers a valuable case study of CE implementation in a developing country, contributing comparative insights into how regulatory evolution interacts with professional motivation in a unique Southeast Asian context.

## Methods

### Study Design

This cross-sectional study involved the distribution of a self-administered questionnaire online and offline from December 2024 to February 2025 to evaluate the attitudes, perceptions, and preferences regarding CE of pharmacists in southeastern Vietnam.

### Eligibility and Sample Size

The inclusion criteria were (1) pharmacists employed in both the government and the private sector in the southeastern region of Vietnam, covering the provinces of Binh Phuoc, Binh Duong, Ba Ria-Vung Tau, Dong Nai, and Tay Ninh as well as Ho Chi Minh City, and (2) those proficient in reading and comprehending Vietnamese. Pharmacists who submitted incomplete questionnaires or provided the same answer (eg, only “A”) throughout the questionnaire were excluded.

The minimum sample size was determined using the formula used by the WHO [20]:


$$N=\frac{{\left(Z\alpha /2\right)}^{2}\times P(1-P)}{d^{2}}$$

where *Z_α/2_* denotes normal distribution with a 95% CI (Z_α/2_=1.96), *d* is an error margin of 0.05, and *P* denotes the proportion of pharmacists with positive attitudes toward CE (*P*=.7, based on the pilot study). To account for potential exclusions, an additional 10% was incorporated into the sample size, resulting in a minimum requirement of 355 participants.

### Data Collection

#### Questionnaire Development

A questionnaire was developed following a comprehensive review of the literature [12,13,15,16,19,21,22], after which it was translated on the basis of conceptual equivalence using a four-step process adapted from the WHO [23]: (1) two independent forward translations, of which the initial translation from the original English version into Vietnamese was performed by a professional translator, (2) a review of the first draft by an expert panel comprising five pharmacists operating in different fields relevant to the study, (3) cognitive interviews performed by 25 pharmacists who did not participate in the main research, and (4) revision based on feedback and suggestions. Face and content validity were determined in steps 2 and 3.

A pilot study was performed on a convenience sample of 30 pharmacists to identify ambiguities and ensure that the questionnaire items would yield reliable data. Revisions were made on the grounds of feedback derived during the pilot study. The data collected during this phase were excluded from the analyses carried out in the main research. The reliability of the questionnaire’s subcomponents was assessed. The Cronbach α coefficients of the subscales on attitudes toward CE (14 items), perceptions regarding CE (4 items), and satisfaction with CE activities (8 items) were 0.841, 0.821, and 0.897, respectively. These values indicate good internal consistency.

#### Questionnaire

The final questionnaire comprised 4 sections (42 items): demographic characteristics (10 items), attitudes toward CE (14 items), perceptions regarding CE (4 items), and preferences for CE types and topics (14 items; Multimedia Appendix 1).

The demographics section was intended to assess sex, year of birth, marital status, education level, years of experience, ethnicity, organizational type, job position, frequency of overtime, and number of CE courses attended. The section on attitudes toward CE was adapted from the Jefferson Scale of Physician Lifelong Learning (JSPLL) [16]. The 14 items were ranked using a 4-point Likert scale ranging from 1=*strongly disagree* to 4=*strongly agree*. The sum of the item scores was used to compute the total attitudinal score, which was interpreted and categorized as follows: a total score ranging from 14 to 28 was regarded as indicative of poor attitudes, a score of 29 to 42 was a reflection of fair attitudes, and a score of 43 to 56 represented good attitudes.

In the section on perceptions regarding CE, statements were rated on a 5-point Likert scale ranging from 1=*strongly disagree* to 5=*strongly agree*. The sum of the item scores was used to compute the total perception score, which was interpreted and categorized into 3 levels: a total score ranging from 5 to 10 denoted poor perceptions, a score of 11 to 15 reflected fair perceptions, and a score of 16 to 20 represented good perceptions.

The section on CE preferences covered pharmacists’ satisfaction with CE activities (8 items) and their interest in CE topics (6 items), as well as the perceived impact of such education on professional practice and knowledge. The items were rated on a 5-point Likert scale (*strongly disagree*, *disagree*, *neutral*, *agree*, and *strongly agree*).

#### Measures

Data were collected using both paper-based and online questionnaires. A combination of convenience and snowball sampling was used. Participant recruitment was conducted systematically through multiple channels to enhance sample diversity:

1. On-site recruitment: research team members directly approached pharmacists at a selected variety of community pharmacies and hospitals across the urban and suburban areas of Ho Chi Minh City and the neighboring provinces of Dong Nai and Binh Duong. At these sites, pharmacists were informed about the study and could immediately participate by scanning a QR code to access the online questionnaire or by completing a paper version.
2. Digital outreach: the survey was disseminated through closed professional social media groups and forums dedicated to Vietnamese pharmacists, accompanied by a post explaining the study’s purpose.
3. Institutional distribution: the questionnaire was distributed via the official mailing lists of partner universities and professional pharmacist associations in the region. The online questionnaire was administered using Google Forms, with access provided via a QR code and a direct link. To maintain data integrity, the platform was configured to prevent multiple submissions from the same device. As part of the snowball sampling approach, all participants, regardless of recruitment channel, were explicitly encouraged to share the survey link with their pharmacist colleagues.

### Data Processing and Statistical Analyses

The initial sample consisted of 550 pharmacists, among whom 42 were excluded because they provided the same response across the questionnaire. The final sample consisted of 508 pharmacists who satisfied the inclusion criteria. The data were entered directly into Microsoft Excel 2019 for cleaning and checking for missing answers.

The SPSS software (IBM Corp) was used to analyze the data. More specifically, descriptive analysis was conducted to determine frequencies and percentages for categorical variables, while means and SDs were calculated for continuous variables. A one-way ANOVA with 1000 bootstrap resamples was carried out to assess differences in the mean attitudinal and perception scores of two or more unrelated groups based on the same continuous dependent variable, with a *P*<.05 considered indicative of statistical significance.

### Ethical Considerations

This study adhered to the ethical principles outlined in the Declaration of Helsinki [24] and received approval from the Scientific Research Ethics Committee at Pham Ngoc Thach University of Medicine (1237/TĐHYKPNT-HĐĐĐ). Informed consent was obtained from all participants prior to enrollment. Participants were informed of their right to withdraw at any time without consequence. Participants did not receive any compensation for their involvement in the study. To ensure confidentiality in the online survey, the study was designed to be fully anonymous; no personally identifiable information (eg, names and IP addresses) was collected. Data were collected via a secure platform with encrypted transmission and stored on a password-protected, encrypted server accessible only to the principal investigators. In compliance with institutional data governance policy, the anonymized dataset will be retained for a period of 5 years following study completion, after which it will be permanently deleted.

### Risk of Bias Assessment

A risk of bias assessment was conducted using the Appraisal Tool for Cross-Sectional Studies (AXIS; Checklist 1) [25] to determine whether bias mitigation strategies were implemented in this study rather than the provision of a numerical rating of bias risk. The assessment consisted of 20 questions directed toward the study’s design, analysis, and reporting processes, with the response options available being “yes,” “no,” or “don’t know.” To enhance objectivity and reduce potential bias, 2 authors independently conducted the assessment.

## Results

### Sociodemographic Characteristics

Table 1 summarizes the sociodemographic characteristics of pharmacists. The majority of the 508 respondents were female (331/508, 65.2%). The most prevalent age group was 25 to 30 years, who accounted for 38.8% (197/508) of the sample. More than half of the pharmacists were employed in enterprises operating in the private sector (339/508, 66.7%). A substantial proportion of them were single (318/508, 62.6%) and had less than 5 years of professional experience (311/508, 61.2%). Most of the pharmacists attended at least 1 CE course (458/508, 90.2%).

**Table 1.** Demographic characteristics (N=508).

	Value, n (%)
Sex	
Male	177 (34.8)
Female	331 (65.2)
Age (y)	
21-24	109 (21.4)
25-29	197 (38.8)
30-34	76 (15)
≥35	126 (24.8)
Marital status	
Single	318 (62.6)
Married	190 (37.4)
Highest level of education	
Elementary or intermediate or college^a^	108 (21.2)
University	360 (70.9)
Postgraduate	40 (7.9)
Ethnic	
Kinh	486 (95.7)
Other	22 (4.3)
Organizational type	
State administrative agency system	166 (32.7)
Nongovernmental organizations	3 (0.6)
Enterprise-private system	339 (66.7)
Job position	
Staff	446 (87.8)
Manager	62 (12.2)
Years of experience	
<5	311 (61.2)
≥5	197 (38.8)
Frequency of overtime (times/wk)	
<4	457 (90)
≥4	51 (10)
Number of CE^b^ courses attended	
Never	50 (9.8)
At least 1 course	458 (90.2)

a Educational levels in the Vietnamese context: elementary=vocational secondary training; intermediate=technical diploma; and college=3-year college degree.

b CE: continuing education.

### Attitudes Toward CE

As shown in Table 2, participants generally held positive attitudes toward CE, with a majority (311/508, 61.2%) demonstrating good attitudes and a mean score of 44.4 (SD 5.5). There was a near-universal agreement (457/508, 89.9% combined) with the statement “I take every opportunity to gain new knowledge/skills.” A notable proportion of respondents reported disengaging from routine professional activities, with 25.2% (128/508) disagreeing that they “routinely attend meetings of pharmacy organizations” and 31.9% (162/508) disagreeing that they “read professional journals at least once every week.”

**Table 2.** Attitudes toward continuing education (CE)^a^.

Item	Pharmacists’ attitude	Likert scale, n (%)				Value, mean (SD)
		1^b^	2^c^	3^d^	4^e^	
A1	Searching for the answer to a question is in and by itself rewarding.	2 (0.4)	33 (6.5)	271 (53.3)	202 (39.8)	3.3 (0.6)
A2	CE is a professional responsibility of all pharmacists.	4 (0.8)	46 (9.1)	263 (51.8)	195 (38.3)	3.3 (0.7)
A3	I enjoy reading articles in which issues of pharmacy are discussed.	2 (0.4)	56 (11.0)	310 (61.0)	140 (27.6)	3.2 (0.6)
A4	I routinely attend meetings of pharmacy organizations.	11 (2.2)	128 (25.2)	267 (52.6)	102 (20.0)	2.9 (0.7)
A5	I read professional journals at least once every week.	26 (5.1)	162 (31.9)	246 (48.4)	74 (14.6)	2.7 (0.8)
A6	I routinely search for computer databases to find out about new developments in my specialty.	11 (2.2)	60 (11.8)	297 (58.4)	140 (27.6)	3.1 (0.7)
A7	I believe that I would fall behind if I stopped learning about new developments in pharmacy.	2 (0.4)	16 (3.2)	251 (49.4)	239 (47.0)	3.4 (0.6)
A8	One of the important goals of Faculties of Pharmacy is to develop students’ lifelong learning skills.	3 (0.6)	32 (6.3)	258 (50.8)	215 (42.3)	3.3 (0.6)
A9	Rapid changes in therapeutics require constant updating of knowledge and the development of new professional skills.	2 (0.4)	13 (2.6)	256 (50.4)	237 (46.6)	3.4 (0.6)
A10	I always make time for self-directed learning, even when I have a busy work schedule and other obligations.	7 (1.4)	89 (17.5)	306 (60.2)	106 (20.9)	3.0 (0.7)
A11	I recognize my need to constantly acquire new professional knowledge.	3 (0.6)	24 (4.7)	269 (53.0)	212 (41.7)	3.4 (0.6)
A12	I routinely attend CE courses to improve patient care.	14 (2.7)	121 (23.8)	263 (51.8)	110 (21.7)	2.9 (0.7)
A13	I take every opportunity to gain new knowledge/skills that are important.	5 (1.0)	46 (9.1)	321 (63.1)	136 (26.8)	3.2 (0.6)
A14	My preferred approach in finding an answer to a question is to search for the appropriate computer databases.	6 (1.2)	47 (9.3)	285 (56.0)	170 (33.5)	3.2 (0.7)

a Poor attitude: 4/508 (0.8%); fair attitude: 193/508 (38%); and good attitude: 311/508 (61.2%). The average total score using bootstrap (1000 times) was 44.4 (SD 5.5).

b 1: strongly disagree.

c 2: disagree.

d 3: agree.

e 4: strongly agree.

### Perceptions Regarding CE

Table 3 shows the findings related to the participants’ perceptions regarding CE. More than half (288/508, 56.7%) held good perceptions, 39.4% (200/508) exhibited fair perceptions, and 3.9% (20/508) showed poor perceptions. Their mean perception score was 15.6 (SD 2.7). The majority of the pharmacists agreed and strongly agreed with the assertion that CE helps increase knowledge (434/508, 85.4%). Over 60% of the respondents agreed or strongly agreed with the remaining statements.

**Table 3.** Perceptions regarding continuing education (CE)^a^.

Item	Pharmacist’s perception	Likert scale					Value, mean (SD)
		1^b^	2^c^	3^d^	4^e^	5^f^	
P1	The value of the employer places on his participation in CE	8 (1.6)	41 (8.1)	136 (26.8)	235 (46.2)	88 (17.3)	3.7 (0.9)
P2	Your interest in/value of CE	6 (1.2)	12 (2.4)	110 (21.6)	283 (55.7)	97 (19.1)	3.9 (0.8)
P3	CE affects the way you practice	9 (1.8)	40 (7.9)	124 (24.4)	239 (47.0)	96 (18.9)	3.7 (0.9)
P4	CE helps increase your knowledge	5 (1.0)	3 (0.6)	66 (13.0)	230 (45.3)	204 (40.1)	4.2 (0.8)

a Poor perception: 20/508 (3.9%); fair perception: 200/508 (39.4%); and good perception: 288/508 (56.7%). The average total score using bootstrap (1000 times) was 15.6 (SD 2.7).

b 1: strongly disagree.

c 2: disagree.

d 3: neutral.

e 4: agree.

f 5: strongly agree.

### Preferences Regarding CE

**Figure 1.**
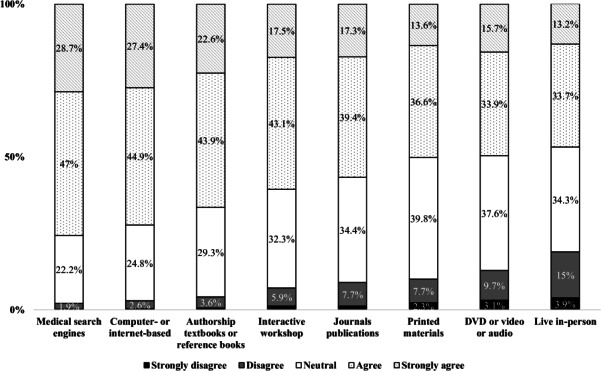
Pharmacists’ level of satisfaction with continuing education activities.

Figure 1 illustrates the participants’ levels of satisfaction with CE activities. The majority preferred computer- or internet-based CE (367/508, 72.2% agreed or strongly agreed with this option) and medical search engines (385/508, 75.7% agreed or strongly agreed with this option). The option of live in-person learning received mixed feedback from participants: 15% (76/508) of the respondents disagreed with the aforementioned options, 34.3% (174/508) exhibited neutrality, and 46.9% (238/508) expressed agreement. The pharmacists showed little preference for DVD- or video- or audio-based learning, with 9.7% (49/508) disagreeing and 37.6% (191/508) showing neutrality with respect to this option. Textbooks or reference books were well received, with 43.9% (223/508) indicating preference and 22.6% (115/508) showing a strong preference for these materials.

Pharmacists' preferences for CE topics were assessed, with the most favored issue being results on skill development (415/508, 81.7% agreed or strongly agreed with this option), followed by innovations in pharmacy practice (399/508, 78.5% agreed or strongly agreed with this option), pharmacy management concepts (398/508, 78.3% agreed or strongly agreed with this option), and innovations in disease management (397/508, 78.1% agreed or strongly agreed with this option). The least preferred topic was innovations in pharmaceutical manufacturing (303/508, 59.6% agreed or strongly agreed with this option).

### Evaluation of Mean Scores for Perceptions, Attitudes, and Impact in Relation to Sociodemographic and Professional Characteristics

The mean attitudinal and perception scores of the pharmacists pointed to significant differences between groups with varying levels of education (43.3, SD 5.4 vs 44.6, SD 5.4 or 45.3, SD 6.0 for mean attitudinal score, *P*=.048 and 15.1, SD 2.6 vs 15.7, SD 2.8 or 15.6, SD 2.5 for mean perception score, *P*=.046; Table 4). Significant differences in mean attitudinal scores were found between respondents categorized by job positions (44.2, SD 5.3 vs 45.7, SD 6.2; *P*=.04) and frequency of overtime (44.2, SD 5.5 vs 45.9, SD 5.0; *P*=.03).

**Table 4.** Comparison of mean attitudinal and perception scores in relation to sociodemographic and professional characteristics.

	Attitude		Perception	
	Mean (SD)	*P* value^a^	Mean (SD)	*P* value^b^
Age (y)		.59		.46
21-24	44.9 (5.9)		15.9 (2.4)	
25-29	44.1 (5.1)		15.6 (2.7)	
30-34	44.7 (5.9)		15.3 (2.7)	
≥35	44.3 (5.3)		15.4 (2.9)	
Gender		.74		.14^c^
Male	44.5 (6.0)		15.3 (3.0)	
Female	44.3 (5.1)		15.7 (2.5)	
Ethnic		.27		.82
Kinh	44.4 (5.4)		15.5 (2.7)	
Other	43.1 (6.7)		15.7 (2.6)	
Marital status		.90		.38
Single	44.4 (5.5)		15.6 (2.6)	
Married	44.3 (5.4)		15.4 (2.9)	
Highest level of education		*.048*		*.046*
Elementary or intermediate or college^d^	43.3 (5.4)		15.1 (2.6)	
University	44.6 (5.4)		15.7 (2.8)	
Postgraduate	45.3 (6.0)		15.0 (2.5)	
Organizational type		.68^c^		.95
State administrative agency system	44.7 (6.2)		15.5 (3.0)	
Nongovernmental organizations	45.0 (4.4)		15.7 (0.6)	
Enterprise-private system	44.2 (5.1)		15.6 (2.6)	
Job position		*.04*		.61
Staff	44.2 (5.3)		15.6 (2.6)	
Manager	45.7 (6.2)		15.4 (3.2)	
Year of experience		.48		.59
<5	44.2 (5.4)		15.6 (2.6)	
≥5	44.6 (5.5)		15.5 (2.9)	
Frequency of overtime (times/wk)		*.03*		.85
<4	44.2 (5.5)		15.5 (2.7)	
≥4	45.9 (5.0)		15.6 (2.7)	
Number of CEs^e^ attended		.52^c^		.55
Never	43.9 (5.0)		15.8 (2.7)	
At least 1 course	44.4 (5.5)		15.5 (2.7)	

a The italicized *P *values are used to denote statistical significance, following conventional academic practice. A *P* value <.05 indicates that the observed difference between groups is statistically significant.

b ANOVA test.

c Welch test.

d Educational levels in the Vietnamese context: elementary=vocational secondary training; intermediate=technical diploma; and college=3-year college degree.

e CE: continuing education.

### Risk of Bias Assessment

The risk of bias assessment indicated a low risk across all evaluated domains (Checklist 1). The study had clearly defined objectives, an appropriate design and sampling strategy, and robust data analysis methods, although potential nonresponse bias was noted.

## Discussion

### Main Findings

This study evaluated the attitudes, perceptions, and preferences of 508 southern Vietnamese pharmacists regarding CE. Most of the respondents reported participation in at least 1 CE course. The majority demonstrated positive attitudes and considerably favorable perceptions of CE. Computer- or internet-based CE and the use of medical search engines were the preferred learning methods of the participants. The mean attitudinal scores varied significantly depending on education level, job position, and overtime frequency. Significant differences in the mean perception scores were found across groups with varying education levels.

### Sociodemographic Characteristics

Most of the pharmacists were aged between 25 and 30 years (197/508, 38.8%), followed by those >35 years (126/508, 24.8%). The mixed methods approach to data collection, that is, the administration of both online and offline surveys, was intended to achieve an age-balanced sample. The online survey streamlined questionnaire distribution, facilitated data management, and enhanced engagement by allowing the respondents to conveniently complete the survey on their phones or computers from various locations. However, online surveys are often more accessible to younger individuals, potentially leading to an age imbalance due to sampling bias, low response rates, and data quality concerns [26,27]. To mitigate these limitations, we administered the survey offline, which also enabled direct interaction with the participants [26] and the provision of clarifications, thereby improving response accuracy and increasing participation among older pharmacists. Combining these methods enabled us to maximize data collection efficiency while ensuring a more representative age distribution.

The majority of the pharmacists hold university degrees. Similarly, Adhikari et al [28] reported that 60% of their pharmacist participants completed university education, whereas Poudel et al [29] indicated that 70% of their respondents acquired college diplomas or equivalents. According to Circular No. 22/2011/TT-BYT in Vietnam Law, most of the main divisions in the pharmacy departments of special-, first-, and second-grade hospitals require pharmacists to have a university degree or higher to practice [30]. Most of the pharmacists in the current work have attended at least 1 CE course, while the remaining individuals may have been newly graduating pharmacists who had not obtained a practicing certificate or taken CE classes. As the field of pharmacy continues to advance globally, pharmacy practitioners feel the need to keep in step with innovations to improve their skills in providing better care to patients [28]. Without CE engagement, pharmacists risk falling behind with regard to new drug developments, treatment protocols, and safety guidelines. This knowledge gap can directly impact the quality of patient care, leading to inaccurate medication counseling, increased potential for prescription errors, and compromised treatment outcomes. High-income countries require pharmacists to pursue CPD to maintain licensure and ensure exceptional patient care—this means frequent and regular participation in CE. As shown by Tjin et al [31], Dutch pharmacists participate in CE for an average of 27.0 hours over 11 months and prefer face-to-face learning (85.5%) over e-learning (13.8%).

### Attitudes Toward CE

The questionnaire used in the present research included closed-ended items taken from the JSPLL [32], which has been used in similar studies conducted in Saudi Arabia [16] and Nigeria [21]. The JSPLL is the first instrument intended specifically to measure lifelong learning among health care providers with supporting psychometric evidence [32]. The majority demonstrated positive attitudes, aligning with the findings of Aldosari et al [16]. Specifically, 61.2% showed good attitudes regarding CE, 38% had fair attitudes, and only 0.8% indicated poor attitudes. These findings are consistent with those of Aldosari et al [16], who reported that 60%, 39%, and 1% of the pharmacists participating in their study showed good, fair, and poor attitudes, respectively [16]. The mean score of 44.4 in the present research pointed to overall good attitudes toward CE among the Vietnamese pharmacists. Specifically, most of them agreed that CE is an essential professional commitment for all pharmacists and that failing to keep up with pharmaceutical advancements could hinder their professional growth. Although the attitudinal distribution was similar to the findings of Aldosari et al [16], the underlying drivers for these attitudes are likely fundamentally different, highlighting the influence of the national context. The Vietnamese context lacks a fully institutionalized framework [3]. Therefore, the similarly favorable attitudes observed among pharmacists in this study are arguably more indicative of a strong intrinsic motivation for professional development. This suggests that, even in the absence of a coercive system, Vietnamese pharmacists personally value lifelong learning. This intrinsic drive is a response to the pressures and opportunities within an evolving national health care system, where rapid advancements and increasing patient expectations create a professional imperative to stay current. This finding underscores that the Vietnamese pharmacist workforce possesses a foundational readiness for CE, which could be powerfully leveraged as the formal system continues to develop. However, the greater proportion of them neither routinely participated in pharmacy organizational meetings nor engaged in weekly readings of professional journals. These results are consistent with the findings of Aldosari et al [16]. Acknowledging the responsibility to engage in CE and lifelong learning is essential for pharmacists to maintain professional competence and deliver outstanding patient care as part of a patient-centered approach. The limited participation of pharmacists in professional organizational meetings and the low frequency of reading professional journals may be attributed to time and cost constraints. In addition, these types of CE may be less prevalent due to the increasing preference for internet-based resources. To solve these issues, professional meetings should be widely promoted at pharmacists’ workplaces and scheduled with suitable time and cost considerations. The availability of professional journals should also be increased to enhance attitudes. In fact, increasing access to professional journals enhances attitudes by providing up-to-date research and expert insights that shape a deeper understanding of the field [33]. This exposure encourages critical thinking and helps professionals refine their practices, fostering more informed and positive attitudes.

Pharmacists with advanced degrees are often exposed to academic environments that promote evidence-based practice, critical thinking, and reflective learning, which enhance their appreciation of CE as an essential component of professional competence [34,35]. They also tend to possess stronger career motivation and clearer professional development goals, fostering a greater intrinsic drive for lifelong learning [36,37]. Their advanced level of education may also provide a stronger knowledge base, reducing uncertainty and fostering a more positive attitude of CE participation as a marker of professional identity and responsibility [38]. Consequently, pharmacists with high educational attainment tend to demonstrate stronger commitment to continuous learning than those with low qualifications [39]. The favorable attitudes among pharmacists in managerial positions align with the findings of Darwish et al [17], who reported greater awareness among pharmacist managers in Jordan. Managers often bear supervisory and decision-making responsibilities, which enhance their recognition of CE as essential for maintaining professional competence and ensuring the quality of pharmaceutical services [35,40]. Moreover, such positions may cultivate stronger intrinsic motivation toward continuous learning, as these individuals perceive CE as a pathway for leadership development and professional recognition [38]. Their access to institutional resources and professional networks could also facilitate participation in CE activities. These findings suggest that interventions to improve attitudes may need to be tailored, for instance, by emphasizing practical benefits for frontline staff and leveraging the advocacy of managerial and postgraduate champions.

### Perceptions Regarding CE

The study found that Vietnamese pharmacists held considerably favorable perceptions of CE, with most agreeing that it increases their knowledge. This aligns with global trends observed in studies from Saudi Arabia [15] and Lebanon [22], suggesting a universal recognition of CE’s core purpose among pharmacists. A notable point of divergence, however, lies in the perceived institutional support. While only 63.5% of the respondents agreed that their employers fully recognize the value of CE, this rate is substantially higher than the rates reported in the earlier international studies [15,22]. This divergence cannot be explained by regulatory mandate alone, as CE is compulsory in all three contexts. Instead, it likely reflects the unique and evolving state of Vietnam’s CE landscape. Unlike the more established systems in other countries, Vietnam’s framework has undergone significant recent reforms aimed at enhancing regulatory enforcement and accessibility [4,14]. This period of active development and the concurrent national focus on upgrading health care professional competency have created a distinctive environment. This has potentially fostered a stronger, more tangible sense among Vietnamese pharmacists that CE is becoming increasingly relevant and valued within their specific professional ecosystem, even if employer recognition is not yet universal.

In Vietnam, most pharmacists recognize CE activities as essential for enhancing their professional knowledge and skills [41]. Similarly, in their identification of pharmacists’ professional learning needs in support of expanded roles in practice, Schindel et al [42] discovered that most of the respondents participate in CE activities out of personal interest or due to job requirements. Interestingly, we found that a considerable number of pharmacists disagreed with the idea that CE affects the way in which these professionals practice. This suggests that pharmacists pursue CE to maintain licensure rather than educate themselves on topics that can be applied to their current work. Therefore, understanding pharmacists’ preferences and ensuring that CE topics align with their practice is a critical mission for policy makers and health care institutions.

A critical finding of this study is the apparent discrepancy between the broadly positive attitudes toward CE and the reported low engagement in specific activities like routine journal reading or organizational meeting attendance. This reflects the practical barriers within the Vietnamese context. The positive attitudes likely capture a genuine desire for knowledge and professional growth. However, the translation of this desire into consistent action may be hindered by significant systemic and personal obstacles. These could include overwhelming clinical workloads and limited paid leave, which restrict time for self-directed learning; financial constraints, where the cost of journal subscriptions or conference fees is prohibitive; a perceived lack of immediate relevance of available content to daily practice challenges; or limited access to resources, particularly for pharmacists in rural or community settings. This gap highlights that fostering positive attitudes is only the first step. For CE to be truly effective, systemic changes, such as providing protected time for learning, subsidizing costs, and ensuring the practical relevance of content, are necessary to enable pharmacists to convert their positive attitudes into consistent professional development behaviors.

### Preferences Regarding CE

Computer- or internet-based and medical search engines were the preferred methods of learning by the Vietnamese pharmacists, as with other studies [16,21]. However, the mean scores for these resources were higher than those derived by Alharthi et al [15], suggesting that pharmacists in Vietnam prefer participating in online CE programs. This preference, in turn, may be attributed to the flexibility of online offerings, allowing pharmacists to complete lessons at their convenience. They also reduce or eliminate program and travel costs, making them more accessible options [41,43]. Economic and financial factors may significantly influence the strong preference for online CE among pharmacists in this study. In resource-constrained settings like Vietnam, traditional in-person CE often entails direct costs such as travel, accommodation, registration fees, and indirect costs related to time away from work. For many pharmacists—particularly those working in rural or underfunded health facilities—these costs can be prohibitive. Online CE offers a cost-effective alternative, eliminating travel expenses and reducing opportunity costs, thereby making professional development more financially accessible. In addition, the increasing availability of free or low-cost online CE programs offered by hospitals, universities, and professional associations further reduces financial barriers. This shift in learning modality can also be interpreted as a response to broader economic pressures. Health care professionals, especially in low- and middle-income countries, often face modest salaries and must finance their own continuing education. These findings contribute to the growing body of literature on digital health education by emphasizing the role of economic factors in shaping learning preferences. From a policy perspective, this highlights the need for targeted investment in digital infrastructure and the subsidization of online CE initiatives. Such measures would not only improve access to education but also help build a more resilient and up-to-date health care workforce. In contrast, Al-Kubaisi et al[12] and other researchers [18,28,44] found that live in-person CE is favored over online learning. This preference stems from the fact that face-to-face classes allow for peer discussions, direct interactions with instructors, and the immediate clarification of questions, which enhance understanding and knowledge retention [28,45].

Most of the pharmacists in our study preferred topics related to skill development, innovations in pharmacy practice, and pharmacy management. These findings are consistent with Iskandar et al.’s study, which showed greater interest in pharmaceutical management than in any other matters [22]. In Dai et al’s research [41], the majority of the participants were inclined to learn about regulations related to drug business operations (75.8%) and pharmaceutical practice (73.5%).

A larger proportion of pharmacists agreed that obtaining certification for a job and commitment to lifelong learning were important reasons for engaging with CE courses. Al-Kubaisi et al [12] found that the main motivation for CE participation among pharmacists is the relevance of a given topic. Therefore, incorporating more engaging and relevant subjects into CE programs can encourage greater participation. The development of future CE programs should focus on comprehensiveness and effectiveness in bridging the divide between theoretical learning and real-world application. CE topics must help pharmacists improve their competencies and abilities as they carry on with their practice. The applicability of what they learn from CE to their work would increase their interest in and motivation for such training. The organization of CE courses must also align with pharmacists’ interests, especially those offered on the web. This goal can be achieved by enhancing engagement and support from employers and by advancing collaborative work between pharmacy regulators and CE providers, which can define the skills and competencies that need to be reinforced and offer CE programs intended for self-directed, lifelong learning.

### Strengths of the Study

This study has several notable strengths. As the first study in Vietnam to explore pharmacists’ attitudes, perceptions, and preferences regarding CE, it addresses a substantial gap in research and delivers novel insights into an underexamined aspect of pharmacy practice. By identifying the key attitudes, perceptions, and preferences that influence pharmacists’ participation in CE, the study derives evidence that policymakers and health care institutions can use to design targeted CE programs that align with pharmacists’ needs and preferences, ultimately enhancing participation rates and improving professional competencies. By offering evidence-based recommendations, this study contributes to the development of more effective CE initiatives, ensuring that they are accessible, relevant, and promotive of pharmacists’ professional growth. Overall, the implication is support for improvements in pharmacy practice and health care quality in Vietnam.

### Limitations

The limitations of this research are likewise worth discussing. First, the use of the JSPLL, which was originally designed to assess physicians’ attitudes toward CE in the United States, may not have fully captured the perspectives of the Vietnamese pharmacists. Although the questionnaire has been validated and culturally adapted for Vietnamese people, the tool may not have reflected unique local professional development needs and contextual factors. The potential impact of this is a measurement bias that could have led to an underestimation of certain attitudes or perceptions highly specific to the Vietnamese pharmacy context. Consequently, some of the nuanced barriers or motivators for CE in Vietnam might remain unidentified. Second, the structure of the questionnaire may have limited the scope of responses, particularly in sections where predefined answer options were provided for CE preferences (eg, reading journal articles, and attending conferences or seminars). This limitation could have constrained the depth of insights into preferred learning methods and emerging CE trends. Third, since the study used a self-administered questionnaire, there was potential for response bias, wherein the participants may have tended to agree with the statements because this was expected or favorable rather than reflecting their true attitudes and behaviors. The likely impact is an inflation of positive scores on attitudinal items, and the prevalence of negative or ambivalent attitudes is likely underreported. Fourth, the study used a web-based questionnaire, which could not ensure that responses were exclusively from the target population, as anyone could access the QR code and complete the form. To address this limitation, a screening question was included to confirm whether the respondent was a pharmacist. The primary mode of data collection was a web-based questionnaire, which, even when combined with paper-based options and on-site recruitment, poses a potential risk of selection bias. The risk of selection bias arises from the fact that internet access is unevenly distributed over the population [46]. Under-coverage occurs because people without internet access are excluded from the survey. It is possible that the sample may still be somewhat skewed toward younger, more technology-comfortable pharmacists who are inherently more receptive to digital surveys. While the mixed mode approach, particularly the on-site paper-based option, was implemented to counter this effect, it cannot be ruled out entirely. Therefore, the generalizability of the findings, especially regarding attitudes toward digital CE, should be interpreted with this potential bias in mind. Fifth, this study was based on quantitative survey methodology. While this approach was effective for identifying and statistically analyzing trends, attitudes, and perceptions across our sample, the closed-ended nature of the questions (including Likert scales and multiple-choice formats) inherently limits the depth of exploration into the underlying motivations, personal narratives, and nuanced contextual factors behind the responses. A qualitative research design would be necessary to provide that deeper layer of understanding. Finally, this study did not examine differences among pharmacists working in various practice settings (eg, community, hospital, and industry). By treating pharmacists as a homogeneous group, it may have obscured significant variations in learning needs, motivations, and perceived barriers. This limits the practical use of the findings for designing targeted CE programs, as the specific, setting-dependent drivers of engagement remain unexplored.

### Future Research

Future research can expand this work by recruiting a nationally representative sample, including pharmacists from various regions of Vietnam, to better capture geographic and demographic differences in CE participation and preferences. Qualitative methods, such as in-depth interviews or focus group discussions, can help researchers more exhaustively illuminate the barriers to and motivators of engagement in CE. Studies should also focus on evaluating the effectiveness of different CE methods, identifying the most impactful learning formats so that they enhance pharmacists’ knowledge, skills, and professional practice. Future studies should investigate the factors that influence these preferences to enable the design of more targeted and effective CE programs. Conducting subgroup analyses across different practice settings is necessary. Investigating the specific CE needs and preferences of pharmacists in community, hospital, industrial, and regulatory roles would provide invaluable data for designing highly targeted and effective CE programs that are responsive to the unique demands of each sector. Finally, longitudinal studies assessing the long-term impact of CE on pharmacists’ career progression, clinical decision-making, and patient outcomes would be valuable in shaping future CE policies and programs.

The findings of this study offer clear, actionable guidance for optimizing CE in Vietnam. While the policies mandate CE, understanding the pharmacists’ views and attitudes toward it is essential for improving both compliance and voluntary participation. For policymakers, the results underscore the need to move beyond a one-size-fits-all regulatory approach. The strong preference for digital and self-directed learning modalities supports the strategic expansion and accreditation of high-quality online CE platforms, which can improve accessibility for pharmacists across diverse geographic and practice settings. For pharmacy educators and training institutions, the high demand for topics in skill development, pharmacy management, and innovative practice indicates a critical need to shift content away from purely theoretical knowledge toward applied, practical competencies. Curricula should be co-designed with practicing pharmacists to ensure relevance. Furthermore, the significant intrinsic motivation observed suggests that promotional campaigns should frame CE not just as a mandatory requirement, but as a valuable tool for career advancement and professional excellence. By aligning program design with these evidence-based preferences, stakeholders can significantly enhance engagement and the overall impact of continuing professional development in Vietnam.

### Conclusions

This study found that although most of the pharmacists exhibited positive attitudes and perceptions regarding CE, a substantial proportion had not participated in CE activities, indicating a gap between awareness and engagement. Variations in preferences for CE formats and topics underscore the need for tailored programs. Addressing participation barriers and aligning CE initiatives with pharmacists’ professional needs may enhance engagement, support professional development, and improve patient care in Vietnam.

## Supplementary material

10.2196/77013 Multimedia Appendix 1 The final questionnaires.

10.2196/77013 Checklist 1 AXIS checklist.

## Abbreviations

AXIS: Appraisal Tool for Cross-Sectional Studies
CE: continuing education
CPD: continuing professional development
JSPLL: Jefferson Scale of Physician Lifelong Learning
WHO: World Health Organization
