# Supplementary material for: Pharmacists’ Attitudes, Perceptions, and Preferences Regarding Continuing Education: Cross-Sectional Study in Vietnam
Source: JMIR Med Educ. 2025 Dec 16;11:e77013. doi: 10.2196/77013 (PMC12707692; doi:10.2196/77013)
Supplement: Multimedia Appendix 1 [file mededu-v11-e77013-s001.docx]

**PART 1. DEMOGRAPHIC CHARACTERISTICS**

|  | **Questions** | **Options** |
| --- | --- | --- |
|  | **Year of birth** | \|___\|___\|___\|___\| |
|  | **Gender** | O 1. Male  O 2. Female |
|  | **Ethnic** | Ο 1. Kinh  Ο 2. Other: ….…………………… |
|  | **Marital status** | O 1. Single  O 2. Married |
|  | **Highest level of education** | Ο 1. Elementary/ intermediate/ College  Ο 2. University  Ο 3. Postgraduate |
|  | **Organizational type** | Ο 1. State administrative agency system  Ο 2. Non-governmental organizations  Ο 3. Enterprise-private system |
|  | **Job Position** | Ο 1. Staff  Ο 2. Manager |
|  | **Years of experience** | Ο 1. < 5 years  Ο 2. ≥ 5 years |
|  | **Frequency of overtime (times/week)** | Ο 1. < 4  Ο 2. ≥ 4 |
|  | **Number of CE courses attended** | ….……………………….…………………… |

**PART 2. ATTITUDES TOWARD CONTINUING EDUCATION**

|  | **Questions** | **Options** | | | |
| --- | --- | --- | --- | --- | --- |
|  |  | **1.**  **Strongly disagree** | **2.**  **Disagree** | **3.**  **Agree** | **4.**  **Strongly agree** |
|  | *Searching for the answer to a question is in and by itself rewarding.* | Ο | Ο | Ο | Ο |
|  | *CE is a professional responsibility of all pharmacists.* | Ο | Ο | Ο | Ο |
|  | *I enjoy reading articles in which issues of pharmacy are discussed.* | Ο | Ο | Ο | Ο |
|  | *I routinely attend meetings of pharmacy organizations.* | Ο | Ο | Ο | Ο |
|  | *I read professional journals at least once every week.* | Ο | Ο | Ο | Ο |
|  | *I routinely search for computer databases to find out about new developments in my specialty.* | Ο | Ο | Ο | Ο |
|  | *I believe that I would fall behind if I stopped learning about new developments in pharmacy.* | Ο | Ο | Ο | Ο |
|  | *One of the important goals of Faculties of Pharmacy is to develop students’ lifelong learning skills.* | Ο | Ο | Ο | Ο |
|  | *Rapid changes in therapeutics require constant updating of knowledge and the development of new professional skills.* | Ο | Ο | Ο | Ο |
|  | *I always make time for self-directed learning, even when I have a busy work schedule and other obligations.* | Ο | Ο | Ο | Ο |
|  | *I recognize my need to constantly acquire new professional knowledge.* | Ο | Ο | Ο | Ο |
|  | *I routinely attend CE courses to improve patient care.* | Ο | Ο | Ο | Ο |
|  | *I take every opportunity to gain new knowledge/skills that are important.* | Ο | Ο | Ο | Ο |
|  | *My preferred approach in finding an answer to a question is to search for the appropriate computer databases.* | Ο | Ο | Ο | Ο |

**PART 3. PERCEPTIONS TOWARD CONTINUING EDUCATION**

|  | **Questions** | **Options** | | | | |
| --- | --- | --- | --- | --- | --- | --- |
|  |  | **1.**  **Strongly disagree** | **2.**  **Disagree** | **3.**  **Neutral** | **4.**  **Agree** | **5.**  **Strongly agree** |
|  | *The value of the employer places on his participation in CE* | Ο | Ο | Ο | Ο | Ο |
|  | *Your interest in/value of CE* | Ο | Ο | Ο | Ο | Ο |
|  | *CE affects the way you practice* | Ο | Ο | Ο | Ο | Ο |
|  | *CE helps increase your knowledge* | Ο | Ο | Ο | Ο | Ο |

**PART 4. PREFERENCES TOWARD CONTINUING EDUCATION**

| **TT** | **Questions** | **Options** | | | | |
| --- | --- | --- | --- | --- | --- | --- |
|  |  | **1.**  **Strongly disagree** | **2.**  **Disagree** | **3.**  **Neutral** | **4.**  **Agree** | **5.**  **Strongly agree** |
|  | *Live in-person* | Ο | Ο | Ο | Ο | Ο |
|  | *Computer/Internet based* | Ο | Ο | Ο | Ο | Ο |
|  | *Interactive workshop* | Ο | Ο | Ο | Ο | Ο |
|  | *DVD/Video/audio* | Ο | Ο | Ο | Ο | Ο |
|  | *Printed materials* | Ο | Ο | Ο | Ο | Ο |
|  | *Journals publications* | Ο | Ο | Ο | Ο | Ο |
|  | *Medical search engines* | Ο | Ο | Ο | Ο | Ο |
|  | *Authorship textbooks/reference books* | Ο | Ο | Ο | Ο | Ο |
|  | *Innovations in disease management* | Ο | Ο | Ο | Ο | Ο |
|  | *Humanities or psychology topic* | Ο | Ο | Ο | Ο | Ο |
|  | *Innovations in pharmacy practice* | Ο | Ο | Ο | Ο | Ο |
|  | *Innovations in pharmaceutical manufacturing* | Ο | Ο | Ο | Ο | Ο |
|  | *Results in skill development* | Ο | Ο | Ο | Ο | Ο |
|  | *Pharmacy management concepts* | Ο | Ο | Ο | Ο | Ο |
