# Supplementary material for: Pharmacists’ Attitudes, Perceptions, and Preferences Regarding Continuing Education: Cross-Sectional Study in Vietnam
Source: JMIR Med Educ. 2025 Dec 16;11:e77013. doi: 10.2196/77013 (PMC12707692; doi:10.2196/77013)
Supplement: Checklist 1 [file mededu-v11-e77013-s002.docx]

The Appraisal tool for Cross-sectional Studies

|  | **Questions** | **Yes** | **No** | **Don’t know/****Comment** | **% Agreement*** |
| --- | --- | --- | --- | --- | --- |
| Introduction | | | | | |
| 1 | Were the aims/objectives of the study clear? | x |  |  | 100% |
| Methods | |  |  |  |  |
| 2 | Was the study design appropriate for the stated aim(s)? | x |  |  | 100% |
| 3 | Was the sample size justified? | x |  |  | 100% |
| 4 | Was the target/reference population clearly defined? (Is it clear who the research was about?) | x |  |  | 100% |
| 5 | Was the sample frame taken from an appropriate population base so that it closely represented the target/reference population under investigation? | x |  |  | 100% |
| 6 | Was the selection process likely to select subjects/participants that were representative of the target/reference population under investigation? | x |  |  | 100% |
| 7 | Were measures undertaken to address and categorize non-responders? |  | x |  | 100% |
| 8 | Were the risk factor and outcome variables measured appropriate to the aims of the study? | x |  |  | 100% |
| 9 | Were the risk factor and outcome variables measured correctly using instruments/measurements that had been trialed, piloted or published previously? |  | x |  | 100% |
| 10 | Is it clear what was used to determine statistical significance and/or precision estimates? (e.g., p values, CIs) | x |  |  | 100% |
| 11 | Were the methods (including statistical methods) sufficiently described to enable them to be repeated? | x |  |  | 100% |
| Results | | | | | |
| 12 | Were the basic data adequately described? | x |  |  | 100% |
| 13 | Does the response rate raise concerns about non-response bias? |  | x |  | 100% |
| 14 | If appropriate, was information about non-responders described? |  | x |  | 100% |
| 15 | Were the results internally consistent? | x |  |  | 100% |
| 16 | Were the results for the analyses described in the methods, presented? | x |  |  | 100% |
| Discussion | | | | | |
| 17 | Were the authors’ discussions and conclusions justified by the results? | x |  |  | 100% |
| 18 | Were the limitations of the study discussed? | x |  |  | 100% |
| Other | | | | | |
| 19 | Were there any funding sources or conflicts of interest that may affect the authors’ interpretation of the results? |  | x |  | 100% |
| 20 | Was ethical approval or consent of participants attained? | x |  |  | 100% |

Note: *Percentage of agreement between two independent reviewers.

Explanation

Question 1: See *Q1* at the beginning of the *Abstract (Objective)* and end of the *Introduction* (last paragraph of Introduction).

Question 2: This study used cross-sectional design relating to point-in-time surveys, which is appropriate for the questions being surveyed. See and evaluate *Q2* at *Methods*.

Question 3: Sample size justification is based on the simplified formula of Yamane. See *Q3* at *Sample size calculation.*

Question 4: The study determined the sample size based on the number of pharmacists in Ho Chi Minh City according to health statistics in 2020. See *Q4* at *Sample size calculation*.

Question 5: The target population of the study was pharmacists in Ho Chi Minh City, the sampling frame included all pharmacists in different pharmaceutical fields. See Q5 at *Study participants and eligibility criteria*.

Question 6: This study provided inclusion and exclusion criteria for sampling representative of the target population. See *Q6* at *Study participants and eligibility criteria*.

Question 7: Since no information was collected from the non-responders in the Google Form survey, no specific measures were undertaken to address or categorize non-responders in this survey. In this study, 10 respondents who chose not to participate and two respondents with incomplete respond were excluded, and no further information or analysis of these non-responders was conducted. This could potentially introduce nonresponse bias, as non-responders may differ in significant ways from those who did participate.

Question 8: *Q8* is used to address the concepts of measurement validity and is specifically aimed to address the appropriateness of the measurements being used. Face validity and content validity were carried out in this study via a four-step translation process of WHO, see content validity at *Step 2* and face validity at *Step 3* in *Tools and procedure*.

Question 9: *Q9* is to gauge the measurement reliability of the study measures. Since the questionnaire was not subject to scoring, an assessment of its reliability was not conducted.

Question 10: The statistical methods, software packages used, and the statistical significance levels were clearly stated. See *Q10* at *Statistics analyses*.

Question 11: This study mentioned variables corresponding to its statistical method. See *Q11* at *Statistics analyses.*

Question 12: All basic and important data was described. See *Q12* at *Results*.

Question 13: The response rate in this study does raise some concerns about potential non-response bias since no measures were taken to assess the characteristics of non-responders. Non-response bias occurs when the individuals who do not respond to the survey differ significantly from those who do, potentially affecting the representativeness of the results.

However, the study addressed non-response to an extent by considering a 10% non-response rate during the sample size calculation (n=389), and the final number of participants analyzed still met the required sample size. Additionally, the use of purposive snowball sampling and multiple distribution channels likely helped mitigate non-response bias by increasing the likelihood of reaching the target population. The approach of asking participants to share the questionnaire with colleagues also broadened the reach, though it does not completely eliminate non-response bias.

Question 14: No information regarding non-responders was collected and described.

Question 15: The numbers given in the results; in the text, figures and tables were checked for consistency by the authors.

Question 16: All results from the analyses mentioned in *Statistics analyses* were described in *Results*.

Question 17: The study presents the main findings of the study, then discusses these in detail and concludes in *Discussion* and *Conclusion*.

Question 18: See *Q18* at *Strengths and weaknesses.*

Question 19: The research was conducted in the absence of any commercial or financial relationships that could be construed as a potential conflict of interest. See *Q19* at *Conflict of Interest*.

Question 20: Participation in the survey was completely voluntary via the electronic consent form on the first page, and anyone can choose not to participate or withdraw by not submitting the response. The research received ethical clearance from the Scientific Research Ethics Committee at the Pham Ngoc Thach University of Medicine (No. 1237/TĐHYKPNT-HĐĐĐ). See *Q20* at *Ethics statement.*
